# Identification of muscle fatigue by tracking facial expressions

**DOI:** 10.1371/journal.pone.0208834

**Published:** 2018-12-18

**Authors:** Marco C. Uchida, Renato Carvalho, Vitor Daniel Tessutti, Reury Frank Pereira Bacurau, Hélio José Coelho-Júnior, Luciane Portas Capelo, Heloiza Prando Ramos, Marcia Calixto dos Santos, Luís Felipe Milano Teixeira, Paulo Henrique Marchetti

**Affiliations:** 1 Department of Adapted Physical Activity, School of Physical Education, University of Campinas, Campinas, São Paulo, Brazil; 2 Department of Physical Education, UNIFIEO, Osasco, São Paulo, Brazil; 3 Department of Physical Education, University of Ribeirão Preto, Guarujá, São Paulo, Brazil; 4 School of Arts Sciences and Humanities, University of São Paulo, São Paulo, Brazil; 5 Institute of Science and Technology, Federal University of São Paulo, São José dos Campos, São Paulo, Brazil; 6 Department of Psychology, Faculty of Americana, Americana, São Paulo, Brazil; 7 Department of Physical Education, University of Sorocaba, Sorocaba, São Paulo, Brazil; 8 Department of Kinesiology, California State University, Northridge, California, United States of America; University of Ontario Institute of Technology, CANADA

## Abstract

Resistance training (RT) is performed at distinct levels of intensity from the beginning to the end of exercise sets, increasing the sensation of effort as the exercise progress to more vigorous levels, commonly leading to changes on the facial expression of RT practitioners. The objective of this study is to evaluate changes in facial expressions using the Facial Action Coding System(FACS) and the activation of facial muscles by surface electromyography(sEMG) at two different levels of effort during resistance exercise and to investigate the correlation between facial expression and exercise intensity and fatigue. Eleven healthy male participants [23±6years; 1.77±6 m; 78±10kg] performed a set of arm curl exercise at 50% and 85% 1RM until muscle fatigue. The Surface electromyography (sEMG activity was recorded simultaneously in areas of the epicranius muscle (EM) and zygomatic major muscle (ZM). Facial expression was recorded and blindly scored by five experienced examiners. Scores (0–5) were based on the level of activity of the ZM (lip corner puller—Action Unit 12-FACS) during exercise. Facial expression and sEMG data were obtained during the exercise at the first repetition and at muscle failure. The root mean square (RMS) of the sEMG amplitude of the EM was significantly increased between the first and last repetition (50%1RM:p = 0.002,d = 1.75; and 85%1RM:p = 0.002,d = 1.54). The RMS values for the ZM were significantly increased between the first and last repetition (50%1RM:p<0.001,d = 2.67; 85%1RM:p<0.001,d = 0.50). The RMS values for the ZM were also increased in 85%1RM compared to values obtained from 50%1RM (p = 0.001,d = 1.12) at the first repetition. AU12 scores and RMS values were not statistically different between 85%1RM and 50%1RM at the last repetition. Furthermore, there was a strong correlation (r = 0.61;*p* = 0.045) between AU12 scores and the sEMG peak for the ZM. In conclusion, changes in facial expression may be directly correlated with different resistance exercise intensities and fatigue.

## Introduction

In the literature, prior investigations observed that facial expression demonstrated during a physical task acts as a non-verbal behavior able to influence the judgment of observers regarding an athlete’s physical effort [1]. More recently, De Morree and Marcora [2, 3] suggest that sports spectators intuitively assumed the effort exerted by athletes based on their facial expression. In this context, inferences can be made about the potential of facial expression to convey physical effort. However, this issue has received little attention from the scientific field.

With respect to the available evidence, most investigations evaluated facial expression via the activity of facial muscles assessed by surface electromyography (sEMG) method, and results have demonstrated that facial sEMG activity increased significantly with increasing workload during aerobic exercise [3], and with power output during an incremental workload cycling test [4].

However, sEMG method may be associated with internal (e.g., cross-talk among muscles, recording of other facial expressions than effort) and external (e.g., high costs, physical space, time-consuming, need of trained experts to assessment and evaluation) limitations [3, 5, 6] restricting its use to facial expression evaluation. Unlike sEMG, facial feature tracking has been considered a valuable method to identify patterns of facial movement relating to emotional states, such as sadness, pain, and effort [7,8, 9]. In this context, a recent seminal study demonstrated that head movement and upper, lower and whole face position—evaluated trough the facial feature tracking—increased during a laboratory cycling trial according to the exercise intensity [10].

Interestingly, resistance training (RT) is performed at distinct levels of intensity from the beginning to the end of exercise sets, increasing the sensation of effort as the exercise progress to more vigorous levels commonly leading to changes on the facial expression of RT practitioners [2]. Therefore, it is possible to argue that facial expression assessment could be used as a tool to measure effort during the execution of resistance exercise because this parameter may change in response to muscle fatigue and exercise intensity [2, 3, 4, 6]. However, for the best of our knowledge, no previous studies aimed at investigating the relationship of facial expression—via facial feature tracking—and physical effort during resistance training.

In addition, although the seminal investigation of Miles et al. [6] had demonstrated interesting results, it is worth mentioning that facial feature tracking was evaluated by artificial intelligence. By contrast, Facial Action Coding System (FACS) method, which was proposed by Professor’s Paul Ekman group [10, 11] has been widely used by psychologists to identify patterns of facial expression possibly associated with emotional states in the clinical practice. This method is useful because does not require electronic gadgets so that facial expression changes are detected by a trained evaluator, who must distinguish facial movements based on a single Action Unit (AU) (corresponding to an individual facial muscle) or a combination of two or more AUs.

Therefore, the objective of this study is to evaluate changes in facial expressions using FACS and the activation of facial muscles (i.e., sEMG) at two different levels of effort in RT and investigate the correlation between facial expression and exercise intensity and fatigue.

## Materials and methods

### Experimental design

This study presents a method to determine muscular effort and fatigue by evaluating the activity of the zygomatic major muscle (ZM) [AU12 (lip corner puller) action and sEMG signals] during arm curl exercise. Moreover, the correlation between FACS-based facial expression and the activation of two facial muscles [epicranius muscle (EM) and ZM] was assessed at two different levels of effort (50% and 85% 1RM) during RT. It is important to mention, that different reasons were attributed to the inclusion of ZM and EM. ZM, which is responsible for the activity of AU12, was studied based on the hypothesis that resistance exercise performed until muscle failure could elicit changes in facial expression similar to those observed in individuals under pain sensation, whose demonstrate drastic changes on AU12 [12]. On the other hand, EM activity has not been associated with pain sensation [13, 11] although prior observations lead us to hypothesize that this muscle could collaborate to the changes observed on facial expression in response to resistance exercise performed until muscle failure. Nevertheless, Huang et al. [9] identified changes on mouth rise (i.e., ZM) and eyebrow (i.e., EM) as key features for perceiving facial expression of effort.

### Participants

Eleven healthy male participants with a mean (± standard deviation [SD]) age of 23±6 years were enrolled in the study. The mean ± SD for height, total body mass and arm curl exercise at 1 Repetition Maximum (1RM) were, respectively, 1.77±6 m; 78±10 kg, and 48.6±7.8 kg. All participants practiced RT for more than 12 consecutive months before the study and were recruited from a university population. In the last six months previous to the enrollment, participants performed RT programs based on multi- and single-joint exercises with multiple sets (~26 sets per session) at moderate-to-high intensities at least 4 times per week to improve strength and muscle mass.

The participants had no musculoskeletal disorders in the past 12 months before the intervention. One year before and during the study, the volunteers did not consume anabolic steroids, ergogenic aids, nutritional supplements or other illegal products known to increase muscle performance.

The Research Ethics Committee of University FIEO approved the study under Protocol No. 029/2011, and all procedures complied with the Helsinki declaration. All participants signed an informed consent form to participate in the study. The individual in this manuscript has given written informed consent (as outlined in PLOS consent form) to publish these case details.

### Procedures

Each subject visited the laboratory in two distinct training sessions separated by at least one week. The participants were instructed to refrain from performing strenuous activities for 48 h before the procedure.

In the first session, the participants signed the informed consent form and became familiar with the procedures used in the second session. After that, weight (0.1kg clinical scale, Filizola), height (0.01m stadiometer, Filizola), and 1RM arm curl exercise [14] were measured to characterize the sample and determine the experimental loads. In the second session, participants were subjected to an exercise session, and data were collected.

#### 1 Repetition maximum (1RM) test

Participants performed a 5-minute light general warm-up on a cycle ergometer followed by a specific warm-up using the arm curl exercise, which consisted of 8–10 repetitions using light load and 3–5 repetitions using moderate load. The test is based on increasing the resistance on subsequent attempts until the participant was unable to complete an attempt, and the 1RM is considered the maximal weight lifted with proper technique. The 1RM load was determined in up to 5 attempts (one repetition each one), with a minimum 3-minute interval between the attempts.

All trials were performed with the participants using the full range of motion [14].

#### Exercise protocol

The arm curl exercise is a single-joint exercise that stimulates elbow flexor muscles (biceps brachii, brachialis, and brachioradialis) commonly used by physical practitioners that aim at improving muscle mass and muscle strength, as well as patients submitted to physiotherapy. This resistance exercise was selected to the present study since the head of the participant remains stable and in the same virtual position across the performance, facilitating the position of the camcorder and the performance of other technical procedures.

The participants were asked to select a comfortable standing position with their feet hip-width apart, arms extended, and hands shoulder-width apart holding a regular barbell (1.2 meters). All participants performed concentric and eccentric muscle contractions with a full range of motion using a barbell and weight plates.

The data collection session began with a warm-up with one set of 12 repetitions of arm curl exercises at 50% 1RM. Subsequently, all participants performed a single set with the maximal number of repetitions of arm curl exercise (until muscle failure) starting at two different intensities (50% and 85% 1RM), with a 10-min rest interval between them. A within-subjects, single-blind counterbalanced design was used to select the exercise intensities (50% and 85% 1RM).

#### Surface electromyography (sEMG) of facial muscles

The facial area of EMG electrodes attachment was shaved and the skin was cleaned with alcohol. Bipolar passive disposable dual Ag/AgCl snap electrodes with a 1-cm diameter for each circular conductive area and 1-cm center-to-center spacing were placed over the longitudinal axes of the EM (involved in raising the eyebrows and wrinkling the forehead) and ZM (involved in raising the mouth corners upwards) in the direction of the muscle fibers. The sEMG activity was recorded simultaneously in both muscles on the right side of the head during arm curl exercise until muscle failure. The reference electrode was placed over the clavicle bone. The sEMG signals of the EM and ZM were recorded using an electromyographic acquisition system (PowerLab, AD Instruments, CO, USA) at a sampling rate of 2000 Hz and were analyzed using commercial software (DATAQ Instruments Hardware Manager, DATAQ Instruments, Inc., OH, USA). The EMG activity was amplified (bi-polar differential amplifier, input impedance of 2MΩ, common mode rejection ratio > 85 dB min (60 Hz), gain ·1000, noise >5 μV) and analog-to-digitally converted (12 bit). A manual trigger was used to define the beginning and end of each complete cycle of joint movement defined by each concentric and eccentric action, and the sEMG activity of both muscles was analyzed in each movement cycle. The criterion adopted to normalize the sEMG data was the maximal voluntary isometric activation (MVIA), collected 10 minutes before physical exercise intervention. Three MVIAs were performed during 3-s maximum simultaneous contractions of the muscles of the forehead and temporomandibular joint, and sEMG data were acquired in both muscles. For the sEMG data, the root mean square (RMS) (150 ms of moving window) of the sEMG amplitude was calculated, and the RMS of the sEMG amplitude of each MVIA was used for normalization. The first MVIA was performed to familiarize the participant with the procedure.

The digitized sEMG data were first band-pass filtered at 20–400 Hz using a fourth-order Butterworth filter with a zero lag. The amplitude of each sEMG signal was expressed as an RMS peak (150 ms of moving window) and normalized by the MVIA. All data were analyzed using a customized program written in Matlab (Mathworks Inc., EUA). The mean RMS peak during the two initial and two final movement cycles was calculated. Only the movement cycles before muscle failure were considered. The test-retest reliability was estimated at the RMS peak using the intraclass correlation coefficient (ICC) and corresponded to 0.83 for the EM and 0.96 for the ZM.

#### Image data acquisition and analysis

**Fig 1:**
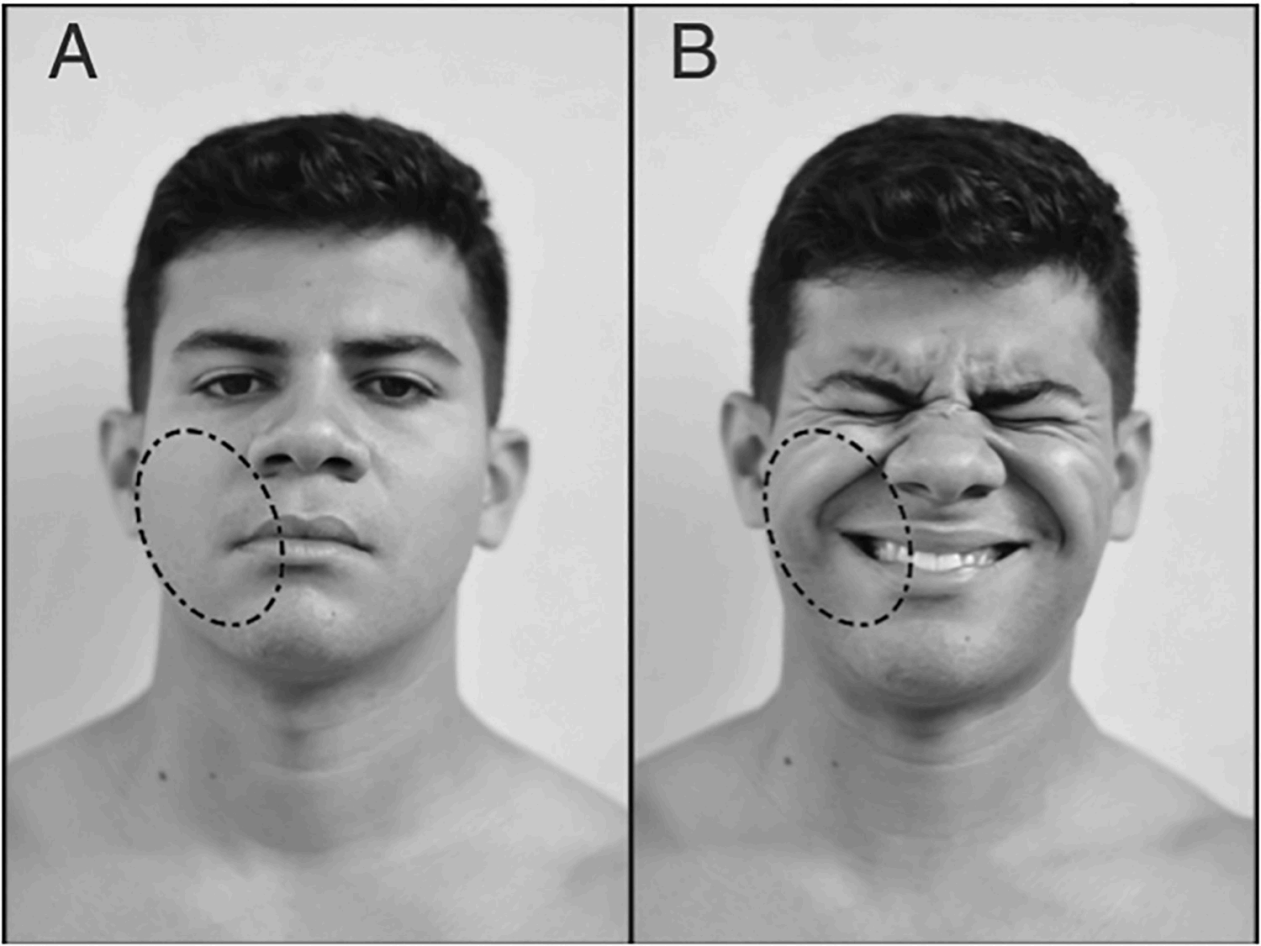
Example of facial expression. Facial expression at rest (A) using a score of zero and in the last exercise repetition until muscle fatigue (B) using a score of five. The oval dash corresponds to the AU12.

**Fig 2:**
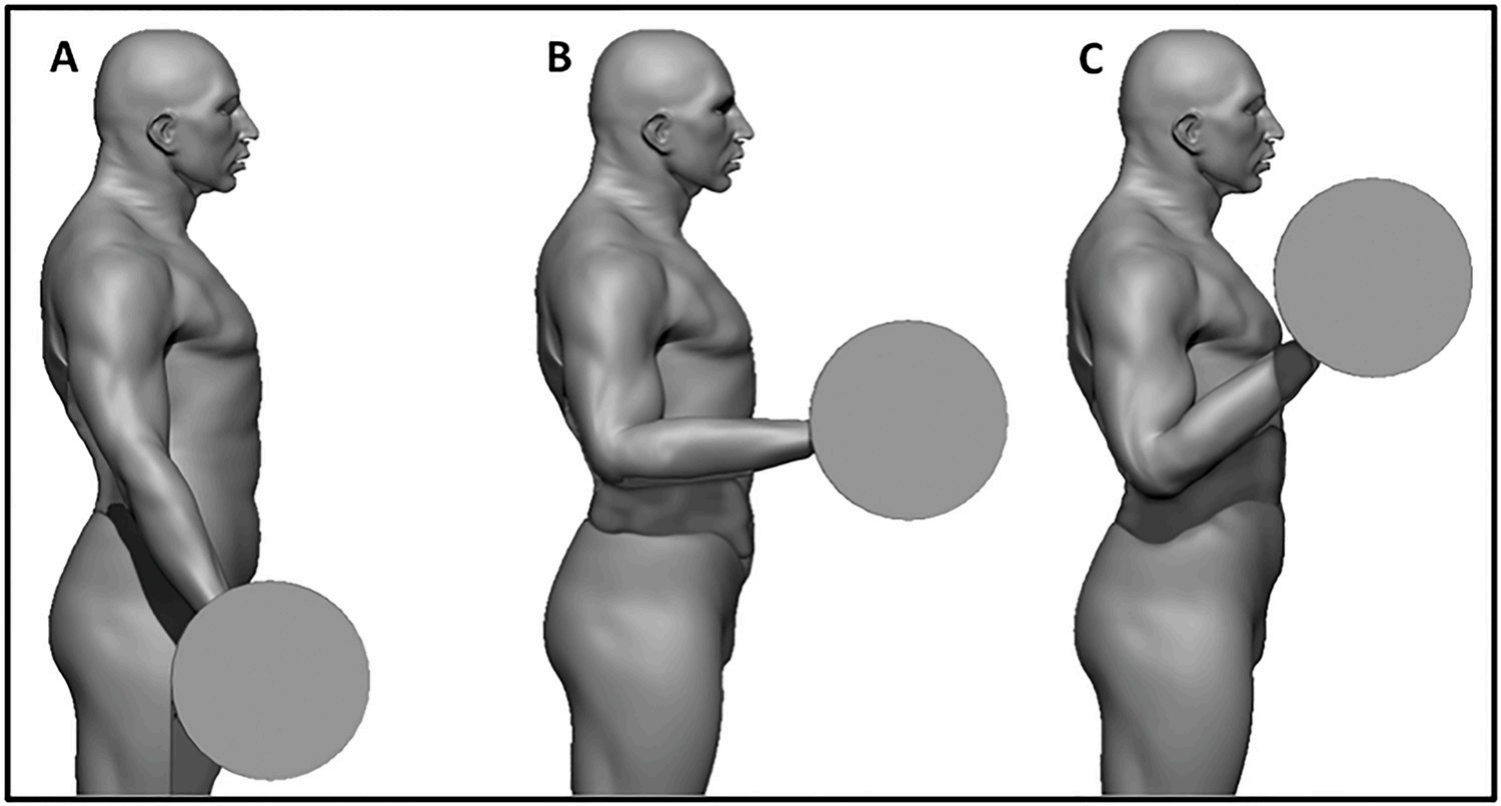
Representation of the execution of the arm curl exercise. A: initial position; B: 90 degrees of elbow flexion, when the AU12 was analyzed and; C: final position.

The images were recorded using a commercial digital camcorder (30 frames per second) connected to a standard computer and positioned 2.3 meters from the subject. The leading researcher instructed the participants to perform the exercises as usual. The full frontal view of the upper body was recorded, but the camcorder focused on the subject’s face. The recordings were performed at the same time of sEMG data collection. Facial expression was analyzed using FACS guidelines and Action Units (AUs). This system can be used to evaluate the activity of several muscles [15]. AU12 corresponds to the ZM, which triggers the lip corner pull movement. The intensity of AU12 was classified using a score from zero to five, adapted from FACS [15], and a score of zero was defined as cases in which there was no evidence of specific AUs and for neutral scoring. The remaining scores indicated the degree of change in facial expression as follows: one, trace change; two, slight change; three, marked or pronounced change; four, drastic change; and five, extreme change (Fig 1). A score of 4 to 5 was used when the lip corners were raised obliquely to make a U shape and the nasolabial furrow deepened, producing an oblique movement of the skin in that area (Fig 1B) [15]. AU12 was monitored during all repetitions but was evaluated in two conditions—first and last exercise repetition (i.e., the last repetition was considered a full range of motion)—during the arm curl exercise at 50% and 85% 1RM. The images were analyzed during the concentric muscle action at 90 degrees of elbow flexion (Fig 2).

For facial expression decoding, five RT researchers (over 10 years RT experience) familiar with AU12 FACS analyses individually tracked the facial expressions of each volunteer. These researchers evaluated the AU12 of each volunteer using FACS’ guidelines and AUs in each repetition until muscle fatigue and scored the volunteers individually and blindly at 50% and 85% 1RM. They were instructed to use FACS as established by Ekman, Friesen, and Hager [15]. A pilot study, six months duration before the initial of this work, was elaborated to offer the learning opportunity, practicing and evaluate using the FACS (UA12). Every two weeks, during this period, there was a research meeting to discuss and calibrate the analysis based on FACS (UA12), resulting in approximately 50 hours of FACS practicing.

Each subject was evaluated five times (not in the same session or day) by different examiners, and each examiner evaluated a maximum of three participants in each session. Facial expressions were analyzed in a dark room where the videos were projected [up to 3 times on a white screen using a light projector (Epson 3LCD)], and each evaluator scored the AU12 intensity during the repetitions with a full range of motion at the elbow joint.

### Statistical analysis

Data normality and variance homogeneity were confirmed using the Shapiro-Wilk test and Levene test, respectively. Two-way analysis of variance (ANOVA) was used to compare the mean differences in the RMS values of sEMG amplitudes between the two levels of intensity (50% and 85% 1RM) and between the first and last repetition. Post-hoc comparisons were performed using Bonferroni correction. The effects of exercise on facial expression were assessed using one-way ANOVA, and Tukey’s post-hoc comparisons were made to determine differences between the two repetitions. The Pearson product-moment correlation coefficient (r) was calculated for the variation (Δ%) between the RMS values (50% and 85% 1RM) and AU-12 values (50% and 85% 1RM) in the first and last repetition. The number of RM between 50% and 85% 1RM was compared using Student’s unpaired *t*-test. Cohen’s formula was used to calculate the effect size (ES) in recreationally resistance-trained participants [16], and the results were based on the following criteria: <0.35, marginal effect; 0.35–0.80, small effect; 0.80–1.50, moderate effect; and >1.5, large effect. The test-retest reliability (ICC) was evaluated using the following criteria: <0.4, poor; 0.4 to <0.75, satisfactory; ≥0.75, excellent [16]. All statistical analyses were conducted using the Statistical Package for the Social Sciences (SPSS) software version 18.0, and p-values smaller than 0.05 were considered statistically significant.

## Results

The RMS peak for the EM was significantly increased between the first and last repetition at each level of intensity: 50% 1RM [*p* = 0.002, *d* = 1.75 (large effect)] and 85% 1RM [*p* = 0.002, *d =* 1.54 (large effect)].

**Fig 3:**
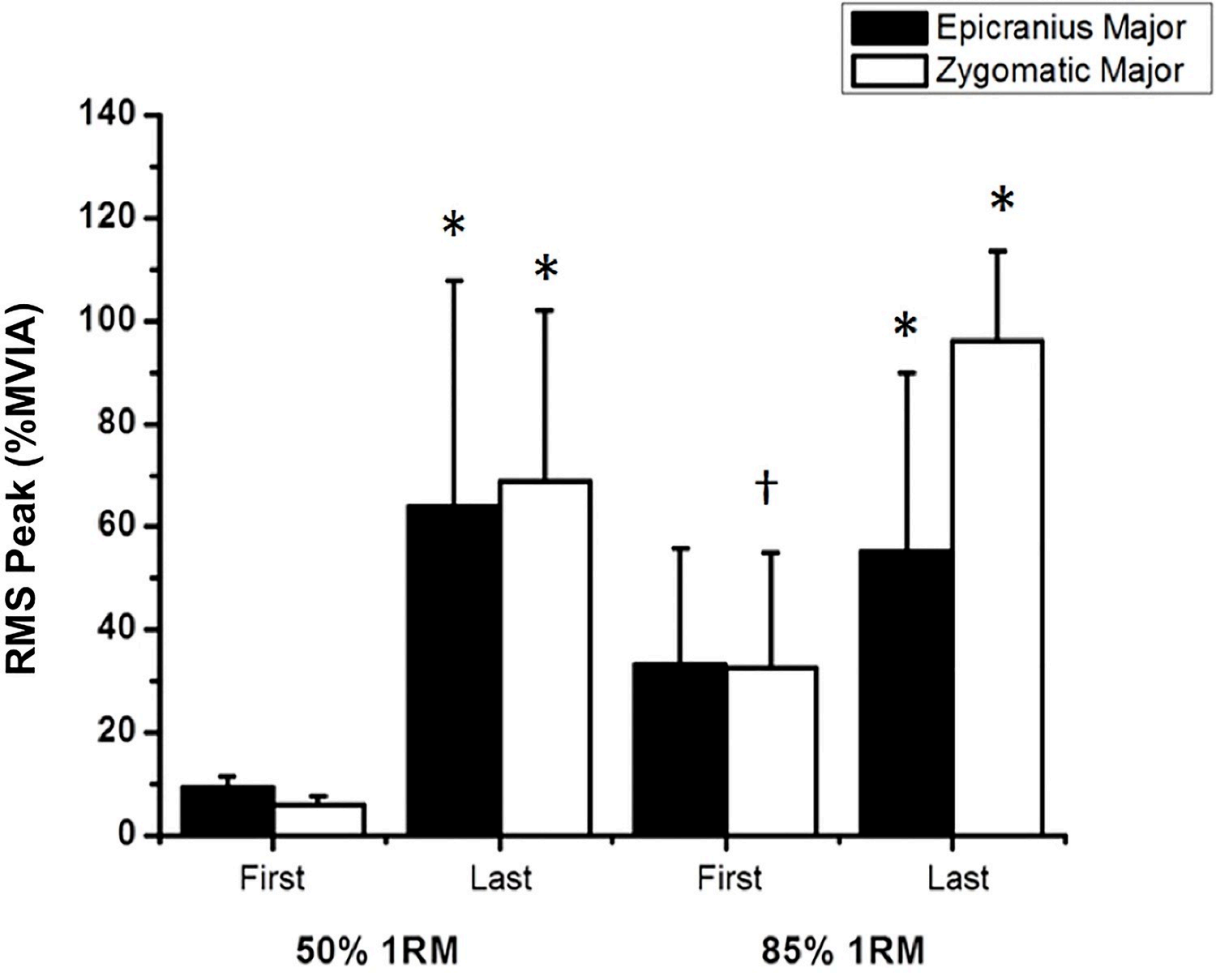
Mean and standard deviation of the RMS peak for the epicranius and zygomatic major muscles during the first and last repetition. *Significant difference between the repetitions (first and last); †Significant difference between the two levels of intensity, *p*<0.05.

Similarly, the RMS peak for the ZM was significantly increased between the first and last repetition at each level of intensity: 50% 1RM [*p*<0.001, *d* = 2.67 (large effect)] and 85% 1RM [*p*<0.001, d = 0.50 (small effect]), and between the two levels of intensity only for the initial condition [*p* = 0.001, *d* = 1.12 (large effect)] (Fig 3).

**Fig 4:**
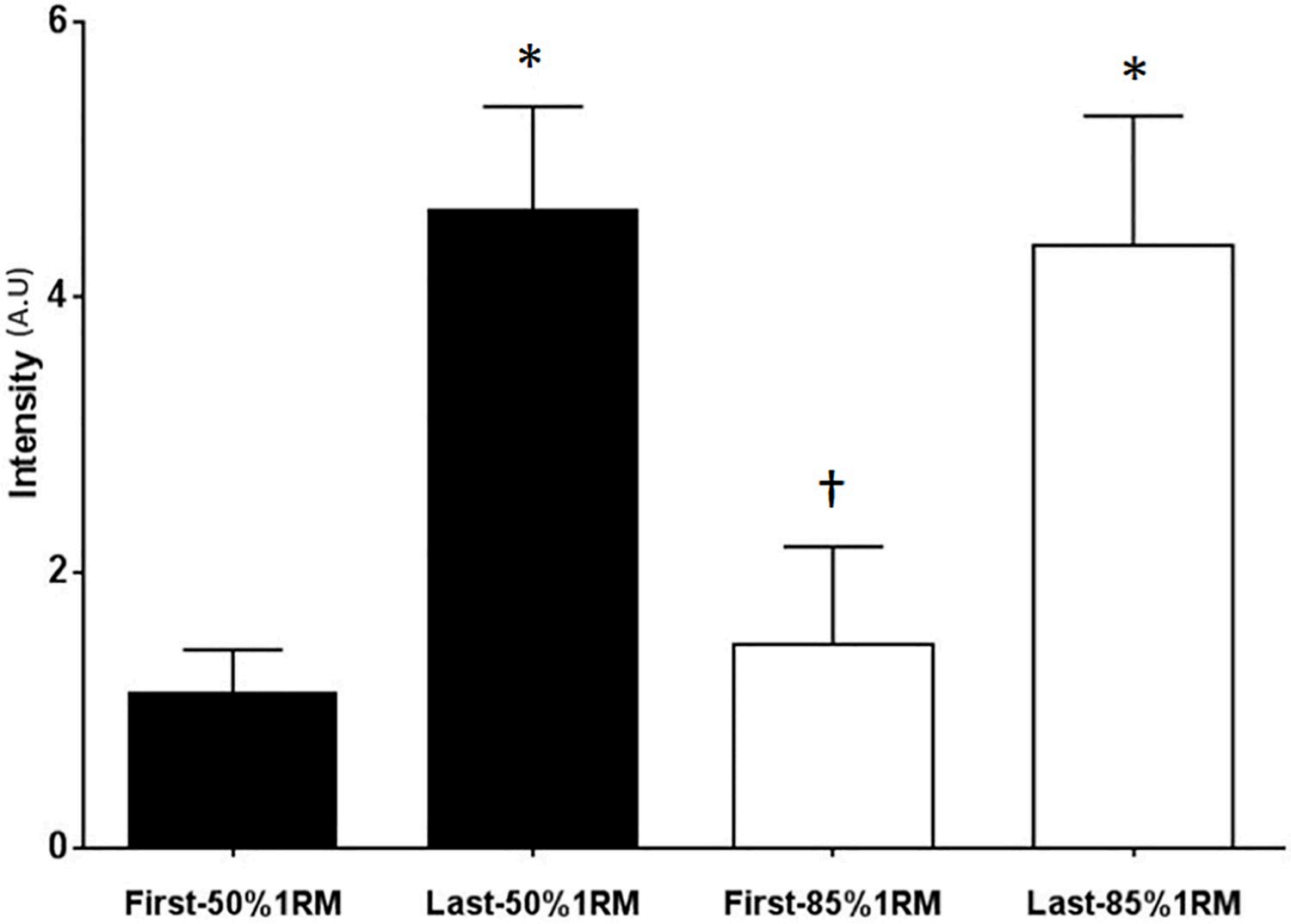
Mean and standard deviation of the AU12 score in the first and last repetition. *Significant difference between the repetitions; †Significant difference between the two levels of intensity, *p*<0.05.

Facial expressions were analyzed using AU12, ZM (lip corner puller) [15], presented in Fig 4. Data were collected in two conditions (first and last repetition of the arm curl exercise) and at two levels of intensity (50% and 85% 1RM). The AU12 results at 50% 1RM were 1.1±0.3 (mean ± SD) in the first repetition and 4.6±0.8 in the last repetition [*p*<0.0001, *d* = 5.79 (large effect)]. The results at 85% 1RM were 1.5±0.7 in the first repetition and 4.4±0.9 in the last repetition [*p*<0.0001, *d* = 4.21 (large effect)]. The AU12 score differed significantly (p<0.0001) between the first and last repetition at each level of intensity, with an increase of approximately 76% and 66% at 50% 1RM and 85% 1RM, respectively. Of note, the AU12 score did not differ significantly between the two levels of intensity in the last repetition (fatigue) but differed significantly between the two levels of intensity in the first repetition (*p* = 0.004).

There was a strong correlation (r = 0.61; *p* = 0.045) between the analysis of facial expression (AU12) and the sEMG peak for the ZM.

Similarly, the number of repetitions until muscle fatigue was significantly different between the two levels of intensity [12.6±2.1 RM (50% 1RM) and 6.6±2.3 RM (85% 1RM)].

## Discussion

The FACS was developed to analyze facial expressions and interpret emotions [15]. In the present study, the analysis of the AU12 indicated a dramatic change in facial expression during arm curl exercise, and this result was strongly correlated with the activity of the ZM. Furthermore, rejecting H0 and accepting the hypothesis of the present study, the activation of facial muscles was significantly associated with changes in the intensity of facial expression and was increased during fatigue compared with the first repetition. These results suggest that the evaluation of a single AU (AU12) by FACS is sensitive enough to indicate changes in the capacity of the skeletal muscle to maintain concentric contraction—muscle fatigue—and may be a valuable tool for monitoring resistance exercise. These results suggest that AU12 assessment by FACS may be a reliable indicator of effort intensity and fatigue. However, more studies are needed to establish the analysis of facial expression as a non-expensive tool for monitoring resistance exercise.

The AU12 defines the contraction of the ZM, which is recruited during smiling and states of happiness [15, 17]. These results were corroborated by Joyal et al. [18], wherein sEMG activity was detected in the ZM during states of happiness. Of note, image analysis indicated that some participants in our sample appeared to smile during maximum effort, actively pulling their lip corners, but exhibited an artificial or forced smile in the last repetition. AU12 is also recruited in painful situations, and some studies used AU12 for assessing pain [7, 12]. The analysis of feelings of pain is complex, and many facial changes occur during short-term acute pain, including lowering the brow, completely closing or narrowing the eyes, raising the cheeks, raising the upper lip, pulling the lip corner obliquely, deepening the nasolabial fold, and opening the mouth and lips at varying levels [19]. Although facial expressions during strenuous exercises are similar to those observed during pain, we believe that the analysis of the latter is much more complex considering the many levels of pain (e.g., low, mild, and intense).

The present study indicates that the activation of facial muscles is correlated with effort intensity during RT. Therefore, muscle activation may not be restricted to the muscles directly involved in the specific task or physical activity, as facial muscles [20].

Our data also reveals differential sEMG and facial expression detected at the first repetition between groups. This result is in agreement with those of De Morree and Marcora [2], who analyzed 20 participants performing leg extensions at four levels of intensity (20, 40, 60, and 80% 1RM) of resistance exercise and observed a significant increase and a positive correlation between the sEMG amplitude and the rating of perceived exertion, and between leg sEMG, task difficulty, and fatigue. During the set of exercises at 50% and 85% 1RM, sEMG activity was increased in the EM and ZM in the last repetition compared with the first repetition. Therefore, there may be a strong correlation between the level of fatigue and the activation of facial muscles, as reported by De Morree and Marcora [2]. However, the activity of the ZM was higher than that of the EM during RT in all study participants. In this respect, the recruitment of cheek muscles during fatiguing contractions may be a more common pattern of facial response than the recruitment of forehead muscles. The analysis of facial expression indicated that AU12 produced remarkable changes in the appearance of the cheek. These changes were easily observed between the rest condition (Fig 1A), that was similar to the first repetition at 50% 1RM, and the last repetition at the two levels of intensity (Fig 1B).

The sEMG results combined with the qualitative analysis of facial expression revealed that the facial expression using AU12 at 50% 1RM was different from that at 85% 1RM in the first repetition. In addition, the FACS scores were higher at 85% 1RM, indicating that facial expression analysis may be a good feature to be used by trained examiners to monitor the participants effort during resistance exercise. In contrast, the sEMG values were similar at 50% and 85% 1RM at the end of each set in the last repetition (repetition maximum). The AU12 results at the two levels of intensity during fatigue are corroborated by the sEMG results and indicate significant changes in facial expressions between the first and last repetition (Fig 1B).

This study has some limitations. First, only young men were evaluated; therefore, the behavior of women, children, and older adults under these experimental conditions remain to be determined. Second, facial expressions were assessed using only AU12; nonetheless, 45 other AUs can be analyzed using FACS, and some of them are being investigated by our research group, included only recreational RT practitioners, the facial expressions of athletes (e.g., weight lifters) and physically inactive individuals have not been assessed to date. Third, sEMG signals and AU12 were only registered in the first and last repetitions, which limit inferences regarding the time-course and repetition response from the first to the last repetition.

Facial expressions are one of the most powerful tools for analyzing non-verbal behaviors associated with emotions and pain. This study, for our knowledge, is the first to correlate the analysis of facial expressions with EMG data during resistance exercise execution. Our results suggest that facial expression analysis has the potential to become, in the future, a tool to differentiate intensities (light [50%1RM] and heavy [85%1RM]) and muscle fatigue in resistance exercise.

Nevertheless, this is a seminal study and our findings are not enough to support the use of FACS in the exercise prescription. Further investigations are still necessary. In our point of view, the main aims of future researchers should include if exercise professionals may identify muscle fatigue through FACS without a camcorder and what is the relationship between FACS and other tools normally utilized by physical trainers professionals, such as RPE. In addition, the application of FACS in combination with other well-established tools should also be considered in future studies.

## Conclusion

In conclusion, changes in facial expression may be directly correlated with different resistance exercise intensities (light and heavy) and fatigue. Our results suggest that facial expressions are in agreement with sEMG increased values at fatigue (repetition maximum) independently of the intensity (50% and 85% 1RM) in resistance exercise.
